# A comparative cost analysis study of pulmonary robotic and video-assisted lobectomy: results of a randomized controlled trial (BRAVO Study)

**DOI:** 10.1590/0100-6991e-20253553-en

**Published:** 2025-07-31

**Authors:** RICARDO MINGARINI TERRA, JULIANA ROCHA MOL TRINDADE, PEDRO HENRIQUE XAVIER NABUCO DE ARAUJO, LETICIA LEONE LAURICELLA, EVELISE PELEGRINELLI ZAIDAN, PAULO MANUEL PÊGO FERNANDESA

**Affiliations:** 1 - Universidade de São Paulo, Instituto do Câncer do Estado de São Paulo, Departamento de Cirurgia Torácica - São Paulo - SP - Brasil; 2 - Universidade de São Paulo Instituto do Coração, Departamento de Cirurgia Torácica - São Paulo - SP - Brasil.

**Keywords:** Robotic Thoracic Surgery, Robotic Surgery Costs, Minimally Invasive Thoracic Surgery, Lung Resection, Lung Cancer, Cirurgia Torácica Robótica, Custos de Cirurgia Robótica, Cirurgia Torácica Minimamente Invasiva, Ressecção Pulmonar, Câncer de Pulmão

## Abstract

**Introduction:**

Robotic thoracic surgery has potential benefits, but the cost is still considered a limiting factor for its wide dissemination in most countries.

**Methods:**

We compared the costs of robotic-assisted (RATS) and video-assisted thoracic surgery (VATS) in the treatment of lung cancer or pulmonary metastasis. Cost analysis was based on micro-costing and individual cost analysis during surgical admission and frequency of services (emergency service, clinic visits, imaging exams, chemotherapy and radiotherapy, reoperation or additional procedures, rehospitalization, and ICU stay) during postoperative 90-day follow-up.

**Results:**

A total of 76 patients were included in this cost analysis (RATS=37, VATS=39). Groups were equivalent in terms of age, gender, comorbidities, and pre-operative status. Total costs of pulmonary lobectomy did not differ between the RATS and VATS groups when considering cost of surgical hospitalization and follow-up of up to 90 days. Mean individual cost per patient in the RATS group was R$35,590.41 (±12,514.97) and R$41,066.98 (±25,891.04) in the VATS group, p=0.564.

**Conclusion:**

Robotic and video-assisted thoracic surgery had similar costs, but longer follow-up studies could be important to demonstrate RATS and VATS costs differences.

## RESUMO

**Introdução:**

A cirurgia torácica robótica tem muitos benefícios em potencial, mas o custo ainda é considerado um fator limitante para sua ampla disseminação em muitos países.

**Métodos:**

Comparamos os custos da cirurgia torácica robótica (RATS) e por videotoracoscopia (VATS) no tratamento do câncer de pulmão e metástases pulmonares. A analise do custo foi obtida pelos dados de microcusteio e de análise de custo individual durante a internação cirúrgica, além da frequencia de uso dos serviços (emergencia, visita hospitalar, exames de imagem, quimioterapia e radioterapia, reoperação ou procedimentos adicionais, re-hospitalização e permanência em leito de terapia intensiva) durante acompanhamento de 90 dias de pós-operatório.

**Resultados:**

O total de 76 pacientes foram incluidos na análise de custo (RATS=37, VATS=39). Os grupos foram equivalentes em relação à faixa etária, sexo, comorbidades e status pré-operaório. Não houve diferença do custo total da lobectomia pulmonar entre os grupos RATS ou VATS quando avaliado custo da internação cirúrgica e seguimento de 90 dias de pós-operatório. O custo médio por paciente no grupo RATS foi de R$35,590.41 (±12,514.97) e R$41,066.98 (±25,891.04) no grupo VATS, p=0.564.

**Conclusão:**

Os custos da cirurgia torácica por vias robótica e videotoracoscópica foram similares, entretando a avaliação de períodos de pós-operatório mais longos seria importante para mostrar diferença de custo entre os procedimentos.

## INTRODUCTION

Lung resection is the gold standard in lung cancer treatment in initial stages, with most cases being recommended for pulmonary lobectomy. Minimally invasive thoracic surgery has shown postoperative benefits, such as reduced postoperative morbidity and reduced hospital stay^1^, without compromising oncological outcomes^2^.

Robotic-assisted thoracic surgery has added relevant technical benefits to minimally invasive surgery, such as better visualization of the surgical field through three-dimensional (3D) imaging and camera control by the surgeon, greater range of motion and better dissection of delicate pulmonary and mediastinal structures, and more precise and tremor-free movements^3^. In addition, the literature has consistently shown equivalent oncologic results for pulmonary resection through robotic (RATS - robotic assisted thoracic surgery) and video surgery (VATS - video-assisted thoracic surgery).

The BRAVO trial, a Brazilian Randomized Controlled study comparing Robotic Assisted and Video Assisted surgery Outcomes in patients undergoing pulmonary lobectomy, analyzed two groups of patients who underwent either robotic-assisted or video-assisted thoracic surgery (RATS versus VATS lobectomy), with comparable demographics, preoperative clinical condition, lung function, and preoperative stage. Postoperative outcomes differed in terms of hospital readmission. The VATS group had a significantly higher number of readmissions in 90 days than the RATS group (RATS 1 vs VATS 8, p=0.029). Additionally, results showed a tendency towards a lower complication rate in 90 days in the RATS group (RATS group 18.9% vs VATS 35.9%, p=0.12) and absence of statistical difference in postoperative pain by the 3rd and 30th days after the procedure. There was also no difference in quality of life reported by the two groups^4^. 

Despite the potential benefits of robotic surgery, the cost is still considered a limiting factor for its wide dissemination in most countries. Implementing a robotic surgery program requires careful consideration of the costs associated with acquiring the robotic platform, doing maintenance, and purchasing supplies, such as tweezers and disposable materials, as well as the cost of specialized training for surgeons and staff to operate the robot. At this study we compare the costs of robotic-assisted and video-assisted surgery in the treatment of lung cancer or pulmonary metastasis in patients included in a randomized controlled study.

## METHODS

This study comprised a cost analysis of patients included in the BRAVO trial, carried out at the Cancer Institute of the State of São Paulo, Brazil, between April 2015 and June 2017. BRAVO was a randomized controlled trial that compared robotic-assisted and video-assisted pulmonary lobectomy (RATS=37 patients and VATS=39 patients, respectively). The primary objective of the BRAVO study was to evaluate postoperative morbidity and mortality up to 90 days in patients undergoing lung lobectomy for lung cancer or lung metastases treatment. The secondary outcomes surveyed were intraoperative complication, length of pleural drainage, length of hospital stay, postoperative pain, readmissions in 90 days, quality of life, and cost comparison.

Patients diagnosed with lung cancer eligible for the study were evaluated using imaging tests, computed tomography and PET-CT. Complementary staging of the mediastinum was performed by EBUS or Mediastinoscopy in cases of tumors larger than 3 cm, central location, or clinical suspicion of nodal involvement through imaging. The study included: (1) patients recommended for lobectomy for lung cancer or lung metastasis treatment; (2) tumors of up to 5cm in diameter; (3) absence of diaphragm invasion, chest wall, mediastinum, or other lobe; and (4) patients evaluated by a pulmonologist and anesthesiologist, with adequate clinical condition for surgery.

The robotic platform used was Da Vinci Si and the surgical technique was the one proposed by Dylewski et al., with three robotic arms and one accessory portal for the physician assistant^5^. VATS lobectomy was performed as standardized in our service, using three portals. Hilar and mediastinal lymphadenectomy was performed only in patients with primary lung cancer. Tubular pleural drainage 28Fr was used in all cases. Patients were referred to the in-patient unit, except for elderly patients with multiple comorbidities or in cases of intraoperative complication; those patients were referred to the ICU after the procedure.

Cost analysis was based on micro-costing and individual cost analysis of 76 patients. The data regarding surgical admission were available in cost spreadsheets grouped by: (1) hospital services; (2) professional services; (3) diagnostic services; (4) materials; (5) orthoses and prostheses; and (6) robotic supplies. Each column represented the specific cost per item and each line represented a patient. “Robot supplies” include robotic clamps, robotic arms cover, and camera. Values were calculated after reviewing import invoices and donations and average amounts were then converted to Brazilian Reais (R$). Costs of postoperative 90-day follow-up data were also provided in terms of frequency of services: emergency service, clinic visits, imaging exams, chemotherapy and radiotherapy, surgery (reoperation or additional procedures), rehospitalization, and ICU stay. Except for surgical procedures, all costs were calculated as the average cost of service in the current year of use. Cost of surgery was calculated individually.

Cost data were collected in US dollars and converted to Brazilian reais at an exchange rate of R$ 3.40, which represents the average monthly exchange rate in the years in which the study was in force (2015 to 2017).

Statistical analyses were performed using SPSS, version 25. Costs are presented as mean plus or minus standard deviation (SD) and the Mann-Whitney test was used to compare groups. All analyses were carried out with a level of significance of p<0.05.

## RESULTS

A total of 76 BRAVO patients were included in this cost analysis (RATS=37, VATS=39). Groups were equivalent in terms of age, gender, comorbidities, and pre-operative status. Postoperative results are better described in a different article, but the relevant results are as follows: RATS operation room time was longer than VATS (241.7±72.6 min vs. 214.4±45.1 min, respectively, p=0.06). There was no difference in ICU stay, chest tube time and in-hospital time, reoperation, complications in 90 days, and 90-day mortality (Table 1). A statistically significant difference was found only in readmissions in 90 days, which were more frequent in the VATS group: 8 VATS patients (20.5%) vs 1 RATS patient (2.7%), p=0.029.

**Table 1:** Postoperative Course.

	Group		
	VATS Group (n=39)	RATS Group (n=37)	P value
ICU time, days (IQ25-75)	0 (0-2)	0 (0-1)	0.99
In-hospital time, days (IQ25-27)	4 (2-5)	3 (2-4)	0.55
Chest tube time days, (IQ25-75)	2 (1-4)	2 (1-2)	0.27
CRP 2POD, n (±SD)	144.6 (±84.7)	100.8 (±86.2)	0.35
Reoperation	2 (5.1%)**	1(2.7%)*	0.59
Complications in 90 days (%)	14 (35.9%)	7 (18.9%)	0.12
Complications ≥ 3 in 90 days (%)	10 (25.6%)	7 (18.9%)	0.58
Readmissions in 90 days, n (%)	8 (20.5%)	1 (2.7%)	0.029
90-day Mortality, n (%)	1 (2.5%)	1 (2.7%)	1.0

ICU: intensive care unit; C-reactive protein at post-operative day 2; *Prolonged air-leak. **Prolonged air-leak1 and empyema1.

**Figure 1:**
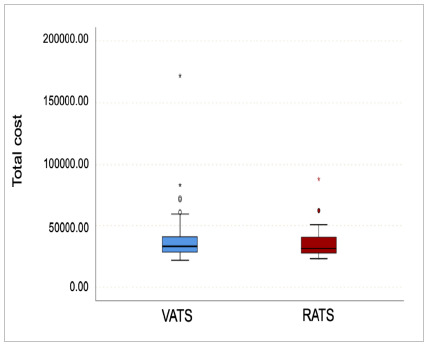
Total costs of pulmonary lobectomy between RATS and VATS groups.

Total costs of pulmonary lobectomy did not differ between the RATS and VATS groups when considering cost of surgical hospitalization and follow-up of up to 90 days. Mean individual cost per patient in the RATS group was R$ 35,590.41 (±12,514.97) and R$41,066.98 (±25,891.04) in the VATS group, p = 0.564, as represented in Figure 1.

Total costs of surgical admission are presented as mean per patient and did not differ between the two groups: VATS= R$ 2,832.86 vs RATS= R$32,522.61, p=0.32). Total costs of surgical admission and costs by category are available in Table 2. The RATS group had a lower cost trend in hospital services, professional services, and diagnostic services, without statistical significance, whereas the VATS group had lower costs for materials (VATS= R$2,024.61 vs RATS= R$2,569.49, p<0.001) and endoscopic staplers (VATS= R$5,595.06 vs RATS= R$ ,023.09, p=0.001).

**Table 2:** Surgical hospitalization costs.

		Group				
		VATS Group n=39		RATS Group n=37		
		Mean	SD	Mean	SD	p value
Surgical Hospitalization	Hospital services	13,349.64	8,590.85	11,209.02	4,154.89	0.035
	Professional services	10,506.88	7,965.87	9,018.55	3,670.71	0.106
	Diagnostic services	1,356.68	2,445.10	1,223.33	1,073.26	0.530
	Materials	2,024.61	2,105.18	2,569.49	616.21	<0.001
	Robot supplies			1,479.13	121.25	NA
	Orthoses and prostheses - staples	1,087.19	1,963.59	1,045.14	716.18	0.401
	Orthoses and prostheses - loads	4,507.87	1,871.50	5,977.95	1,825.95	0.001
	Orthoses and prostheses - staples + loads	5,595.06	3,159.52	7,023.09	1,861.44	0.001
	Total costs (without robot supplies)	32,832.86	23,402.25	31,043.48	9,632.34	0.988
	Total costs	32,832.86	23,402.25	32,522.61	9,638.37	0.321

NA: not analyzable, SD: Standard deviation

Total 90-day follow-up costs were higher in the VATS group (median VATS= R$2,717.25 vs RATS= R$1,545.76, p=0.035).

## DISCUSSION

Our study is the first controlled randomized study to incorporate cost analysis with a few benefits: both VATS and RATS procedures were performed in a single hospital, and we could examine the cost analysis using a single payment source. Our institution is publicly funded and professional services in perioperative time are standardized, therefore, there is no variation in source of payment. 

Minimally invasive lobectomy by video-assisted or robotic assisted surgery is safe and feasible. A meta-analysis published in 2017 by Emmert et al. concluded that RATS and VATS are comparable in terms of perioperative outcomes, with a superiority of RATS in terms of length of hospital stay and chest tube drainage^1^. Our study comprised an initial experience and our results showed no difference between the two approaches in terms of length of hospital stay, chest tube days, and quality of life. The same outcomes were related in other initial experiences^6^ ^,^ ^7^. We expected that a high volume of robotic procedures could measure those differences.

Parameters that comprise cost evaluation are not standardized in the literature, but costs that should be considered when developing a robotic program may include platform acquisition, maintenance, and additional consumables^8^. Following Nasir et al.’s cost definition^9^, we compared only direct cost, which consists of operating room disposable equipment, disposable supplies, staplers, pharmacy items, medications, as well as salaries and benefits of staff caring for the patients. Indirect costs, such as the Da Vinci Si purchase, maintenance, and depreciation, were not considered because the robotic platform was used to develop other specialty robotic programs in our hospital, interfering in cost distribution. 

Even though the majority of studies observed RATS lobectomy as a more expensive approach^10^ ^,^ ^11^, mainly initially, we had similar hospitalization costs between RATS and VATS lobectomy. Deen et al. showed the same results when comparing open RATS and VATS lobectomy, after costs related to depreciation of the robot and robotic-specific supplies were removed^12^. Interestingly, Dylewski-Lazzaro showed, in their retrospective analysis, cost savings of US$560 per case. In our analysis, during surgical admission, RATS material costs were higher due to staples’ loads and disposable robotic supplies, but it was balanced by the cost of VATS patients who had more complications and thus longer ICU and hospital stays. Costs of 90-day follow-ups were not reported in other studies in the literature. We observed that, when following these patients for a longer period, the differences between RATS and VATS lobectomy costs become significant, with a reduction in the cost of RATS lobectomy compared to VATS, correlated with fewer complications. Other studies can confirm those results.

Instead of incremental costs of the robotic platform, some authors suggest that costs could be offset by reductions in postoperative costs (hospital stay, complications, and readmission costs) and by better productivity if patients recover more rapidly and return to work activities^14^. Moreover, it should be considered that price tends to decrease if other robotic platforms and robotic surgical devices manufacturers are present in the market. 

There were some limitations to our study. We compared the costs of only two VATS and thoracotomy approaches since we did not include open pulmonary lobectomy in this study. Although we had more than the 20 cases recommended to complete an adequate training, our study population comprised groups with a small number of patients. Furthermore, a multicenter cost study comprising a larger number of surgeries would be difficult since hospitals have different accounting procedures and could be not comparable.

## CONCLUSION

In conclusion, robotic and video-assisted thoracic surgery had similar costs. Fewer complications and early hospital discharge in the RATS group contributed to lower hospital and professional costs; however, robotic related materials still impacted final costs. Longer follow-up studies could be important to demonstrate RATS and VATS costs differences.
